# Concentration-dependent effects of transforming growth factor β1 on corneal wound healing

**Published:** 2011-11-02

**Authors:** Lingyan Wang, Chun-Ying Ko, Erin E. Meyers, Benjamin S. Pedroja, Nadia Pelaez, Audrey M. Bernstein

**Affiliations:** Mount Sinai School of Medicine, Dept of Ophthalmology, New York, NY

## Abstract

**Purpose:**

There is an unmet challenge to promote wound healing in non-healing wounds such as in the post-LASIK (laser-assisted in situ keratomileusis) cornea. Using human corneal fibroblasts (HCFs) in cell culture, we investigated the concentration dependence of the growth factor transforming growth factor β1 (TGFβ1) on wound closure. Although high concentrations of TGFβ1 leads to scarring, we asked whether low concentrations of TGFβ1 could promote wound healing without generating a large fibrotic response.

**Methods:**

HCFs were cultured in supplemented serum-free media (SSFM). Cell migration was assessed by scratch-wounding. SMAD 2/3 and p38 mitogen-activated protein kinase (p38MAPK) localization and α-smooth muscle actin (α-SMA) organization were evaluated by immunocytochemistry. Active TGFβ was quantified using a luciferase bio-assay.

**Results:**

We found that neutralizing antibody to TGFβ1 reduced cell migration by 73%, compared to immunoglobulin G (IgG) control, establishing that endogenous TGFβ1 (determined to be 0.01 ng/ml) is necessary to promote cell migration. To evaluate the concentration-dependent effects of TGFβ1 on wound closure, HCF migration was quantified to determine the impact of increasing concentrations of TGFβ1 (0.01–1.0 ng/ml). Compared to control (cells in SSFM), the higher concentrations (0.1 and 1.0 ng/ml TGFβ1) significantly decreased cell migration (63%–86%), induced myofibroblast differentiation (83%–88%), increased SMAD 2/3 localization into the nucleus (72%–79%) and inhibited the activation of p38MAPK (51%–63%). In contrast, addition of the lower concentration of TGFβ1 (0.01 ng/ml TGFβ1) promoted a cell migration rate that was similar to endogenous TGFβ, reduced SMAD 2/3 nuclear localization, and stimulated p38MAPK activation. A TGFβ1 blocking antibody and the p38MAPK inhibitor, SB202192, was used to demonstrate that p38MAPK activation is necessary for TGFβ1-induced cell migration.

**Conclusions:**

Together, our data demonstrate that low concentrations of TGFβ1 promote p38MAPK activation that is a key to HCF migration, suggesting that a low concentration of TGFβ may be useful in treating non-healing corneal wounds.

## Introduction

The identification of signaling pathways that promote fibroblast migration into a corneal wound to promote healing without a fibrotic response is an essential area for study. In a normal wound healing response, resident keratocytes are activated to become fibroblasts and myofibroblasts. Activated resident corneal fibroblasts and bone marrow derived fibrocytes migrate into the wound site [1]. The fibroblast-secreted proteases remodel damaged extracellular matrix (ECM) and secrete new ECM that acts as “glue” sealing the wound [2,3].

After laser-assisted in situ keratomileusis (LASIK), the central flap region is not repopulated with stromal cells and the cornea remains unhealed [4,5]. This results in a dramatic decrease in corneal tensile strength [6,7]. Weakening of the cornea after LASIK has been linked to corneal ectasia whereby the post-LASIK cornea exhibits collagen fibril thinning and decreased interfibril distance [8]. Furthermore, because the central cornea remains acellular, there is an increased risk for corneal edema [4,5]. Although these defects occur in a small percentage of LASIK patients, they are potentially severe complications that can lead to loss of vision and may become a greater public health issue with the aging of the population who have LASIK corneas.

To advance our understanding of the role of transforming growth factor β (TGFβ) in wound healing, we have investigated the concentration dependence of TGFβ to wound closure in vitro. A dual role in wound healing has been proposed for TGFβ: It promotes fibroblast cell proliferation and cell migration necessary to repopulate wounded tissue, however it also generates adherent myofibroblasts, which aid in wound closure by contracting wounded tissue but their persistence in a healing wound leads to scarring. Thus, anti-TGFβ antibodies that neutralize TGFβ, significantly reduce myofibroblast differentiation and scarring [9], however, they also inhibit cell repopulation [10,11]. These data suggest that TGFβ promotes wound healing and that TGFβ’s divergent actions may be concentration dependent.

In corneal stromal epithelial and endothelial cells, activation of the p38 mitogen-activated protein kinase (p38MAPK) pathway after wounding is key to increased cell migration that is necessary for wound closure [11-13]. In an effort to identify conditions that promote regenerative healing in the corneal stroma, we investigated the relationship between TGFβ1 concentration and human corneal fibroblast (HCF) cell migration, wound closure, activation of p38MAPK and SMAD 2/3 pathways in vitro. After evaluating a range of concentrations, we determined that addition of 0.01 ng/ml TGFβ most closely resembled the activity of endogenous TGFβ for promoting cell migration, wound closure, and p38MAPK activation without generating a large fibrotic response.

## Methods

### Antibodies and reagents

Transformed mink lung epithelial cells (TMLC) containing the plasminogen activators inhibitor-1 (PAI-1) promoter fused to the luciferase gene were a generous gift of Dr. Daniel Rifkin, New York University, New York, NY. SMAD 2/3 antibody was from Santa Cruz Biotechnology (sc-133098; Santa Cruz, CA), α-smooth muscle actin (α-SMA) antibody was from Sigma (clone 1A4; St. Louis, MO). P38MAPK antibody (ab31828) and Phosph-p38MAPK antibody (ab32557) and TGFβ1 antibody (ab10517) was from Abcam (Cambridge, MA). Secondary Alexa-488 was from Jackson ImmunoResearch (West Grove, PA). Immunoglobulin G (IgG) Antibody was from Jackson ImmunoReserach. TGFβRI inhibitor, SB431542 and p38MAPK inhibitor, SB202190, was from Tocris Bioscience (Ellisville, MO). Bovine collagen (PureCol) was from Advanced Biomatrix (San Diego, CA). TGFβ1 was from R&D Systems (Minneapolis, MN).

### Preparation of human corneal cells

HCF were derived from the stroma of human corneas that were not suitable for transplantation (obtained from National Disease Research Interchange (NDRI), Pittsburgh, PA). Stromal keratocytes were isolated as previously described [14]. To produce fibroblasts, freshly isolated corneal stromal keratocytes were cultured in DMEM-F12 with antibiotic antimycotic (ABAM) and gentamicin (all from Sigma) plus 10% fetal bovine serum (FBS, Atlanta Biological, Lawrenceville, GA). All experiments were done on 10 μg/ml bovine collagen I (Advanced Biomatrix) in supplemented serum-free media (SSFM: DMEM/F-12, 1X RPMI-1640 Vitamin Mix, 1× ITS Liquid media supplement (5 μg/ml each of insulin, transferrin, and 0.05 μg/ml sodium selenite), 1 μg/ml glutathione; (all from Sigma), 1 mM sodium pyruvate, 0.1 mM MEM non-essential amino acids; (all from Gibco-BRL) with ABAM (antibiotic antimycotic, Sigma) and gentamicin (Invitrogen, Carlsbad, CA).

### Migration assay

HCFs were seeded at confluency (1×10^5^ cells/well) on 10 μg/ml collagen in SSFM. The next day cultures were scraped wounded with a 200 μl pipette tip, medium was replaced. After an incubation at 37 °C for 24 h, migration was assessed with T-Scratch software developed by Koumoutsakos group (CSE laboratory), at ETH, Zürich, Switzerland. Briefly, images taken at 24 h for each treatment are imported into this application. This software determines a percent wound closure (the space remaining in the scratch wound) compared to the control (time zero after wounding). For inhibition studies we added 2.5 μg/ml TGFβ1 antibody, 2.5 μg/ml matched IgG control, 10 μM SB202192, or 10 μM SB431542.

### Immunocytochemistry

Cells were fixed with 3% p-formaldehyde (Fisher Scientific, Fair Lawn, NJ) in PBS for 15 min at RT and permeabilized with 0.1% Triton X-100 for 1 min at RT. After blocking non-specific binding with 3% normal mouse serum (Jackson ImmunoResearch), cells were incubated with anti-α-SMA antibody or anti-SMAD 2/3 antibody or anti-p38MAPK antibody followed by Alexa-488 secondary. After washing, coverslips were mounted on slides for viewing with a Zeiss Axioskop microscope and images were captured using a Zeiss Axioscope with a SPOT-2 CCD camera (Diagnostic Instruments, Sterling Heights, MI) and processed by Adobe Photoshop (Adobe, San Jose, CA) software. Photoshop images were exported into the MetaMorph image analysis software package (version 6.3r3; Molecular Devices, Sunnyvale, CA) to determine relative cell area.

### TGFβ activity assay

This bioassay detects active TGFβ. HCFs were co-cultured with TMLC containing the PAI-1 promoter fused to the luciferase gene. Extracellular TGFβ binds to its receptors and signals intracellularly to activate the PAI-1 promoter. HCFs and TMLC were plated together, each at 1×10^5^ cells per well in 24 well dishes in DMEM, 10% FBS, 1 mM L-glutamine with antibiotics. After 24 h the media were replaced with (0.1% BSA in DMEM) and further incubated for 24 h. Luciferase activity was measured using the Bright-Glo detection system (Promega, Madison, WI) and luminescence was determined using a Synergy 2 multi-mode Microplate Reader (Biotek, Winooski, VT). Addition of known amounts of recombinant human TGFβ1 (R&D Systems) to TMLC cells was used to generate the standard curve.

### BrdU staining (data not shown)

Proliferation assays were attempted using two methods. First, HCFs were seeded at low density on collagen in 100 mm plate in either SSFM alone or with increasing concentrations of TGFβ (0.01, 0.1, or 1.0 ng/ml TGFβ). After 24 h 10 μM BrdU (Cell Signaling Technology, Beverly, MA) was added for 4 h before fixation with methanol at −20 °C for 10 min. DNA was then denatured in 2 M HCL for 1 h and integrated BrdU was detected by anti-BrdU monoclonal antibody (Cell Signaling Technology) followed by FITC-conjugated goat anti- mouse secondary antibody (Jackson ImmunoResearch) for 30 min and counter stained by propidium iodide. Slides were evaluated using Zeiss Axioscope with a SPOT-2 CCD camera (Diagnostic Instruments). In the second method, HCFs were seeded at confluency on collagen in a 100 mm plate in SSFM. After 24 h, cells were scratch wounded (using a grid to ensure an equal number of scratches per plate) in the presence of either SSFM or SSFM with increasing TGFβ concentrations (as above) with 10 μM BrdU. After 4 h, cell were fixed and stained for BrdU as above. Differences between conditions were not observed using either technique.

### Activation of p38MAPK by western blot

HCFs were seeded at confluence 2×10^6^ on collagen in SSFM. The next day cells were scratch wounded using a grid to produce consistent wounding per plate. Media was exchanged and reagents were added. After 4 h, cells were lysed in RIPA buffer (50 mM Tris, pH 7.4, 150 mM NaCl, 0.1% SDS, 0.5% sodium deoxycholate, 1% Triton) with protease inhibitors (Roche Applied Scientific, Indianapolis, IN) and the phosphatase inhibitors (HALT; Thermo Scientific , Rockford, IL tific). Lysates were western blotted for p38MAPK and phosph-p38MAPK. Ratios of P-p38MAPK/p38MAPK are graphed.

### Statistical analysis

Standard error between experiments was calculated. All experiments were repeated at least 3 times. P-values were calculated using the students *t*-test. *p-value <0.05, **p-value <0.01, ***p-value <0.001.

## Results

### Neutralizing TGFβ activity inhibits cell migration

Endogenous TGFβ is increased in cells at the wound edge of wounded corneal fibroblasts in vitro [15]. To confirm that endogenous TGFβ is necessary for HCF migration, after scratch-wounding of confluent cells, we blocked TGFβ by adding neutralizing TGFβ1 antibody or matched IgG (control). Neutralizing antibody inhibits total TGFβ1 activity since it binds latency-associated peptide (LAP)-TGFβ preventing the generation of new TGFβ1 as well as binding active TGFβ [16]. A caveat to this experiment is that LAP may contribute to cell migration (unrelated to activation of TGFβ) [17], however, this has not been demonstrated in HCFs.

**Figure 1:**
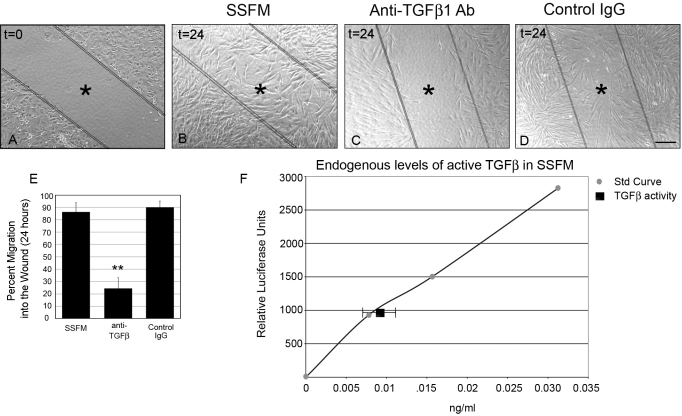
Endogenously secreted TGFβ1 promotes wound healing. HCFs were seeded on collagen in SSFM at 1×10^5^ cells/ml in a 24 well dish. The next day cells were scratch-wounded and imaged (**A**) time zero, or incubated for 24 h with either (**B**) SSFM (**C**) 2 μg/ml anti-TGFβ1 antibody or (**D**) 2 μg/ml matched IgG control. Bar=200 μm, *inside lines denoting wounded area. **E**: Using T-Scratch software, percent cell migration into the wound margin at 24 h compared to time zero was calculated. Each condition was compared to SSFM, **p-value <0.01. **F**: To determine endogenous levels of TGFβ, HCFs were co-cultured with TMLC, which contain the PAI-1 promoter fused to the luciferase gene. This assay demonstrated that HCFs have 0.01 ng/ml active TGFβ. Experiments were repeated at least three times with similar results.

HCF migration was visualized by comparing time zero after wounding (Figure 1A) to 24 h post-wounding (Figure 1B-D). To eliminate the contribution of serum components to cell migration, all studies were performed on HCFs grown under supplemented-serum free conditions (SSFM, see methods) on collagen. These data show that under SSFM conditions, at 24 h, HCFs have almost completely repopulated the wound area (86%; Figure 1B) whereas, the anti-TGFβ1 antibody inhibited cell migration by 73% (Figure 1C) compared to the IgG control (Figure 1D). Percent migration into the wound compared to time zero was quantified using T-Scratch software (see methods; Figure 1E). Because TGFβ neutralizing antibody inhibited cell migration, we sought to determine the levels of active TGFβ in HCFs when plated in SSFM using the sensitive TMLC bioassay (see methods). TMLCs contain the PAI-1 (plasminogen activator inhibitor-1) promotor linked to the luciferase gene and TGFβ activates PAI-1 [18]. We have co-cultured TMLC cells with HCFs to quantify TGFβ activity in HCF in unwounded cells [19]. Using this assay we determined that HCFs under SSFM conditions have 0.01 ng/ml active TGFβ (either secreted or localized to their cell surface; Figure 1F). This assay does not distinguish between TGFβ isoforms however, TGFβ1 neutralizing antibody reduced active TGFβ to undetectable amounts (data not shown), suggesting that this is a main isoform.

### Increasing TGFβ1 concentration correlates with decreasing cell migration

**Figure 2:**
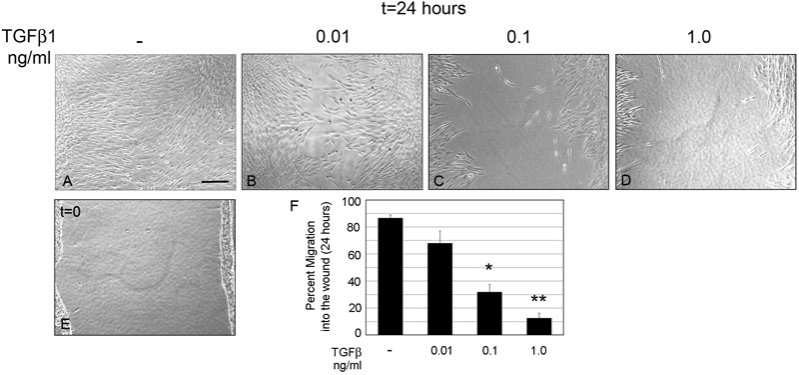
Increasing TGFβ1 concentrations reduces HCF migration. HCFs were seeded on collagen in SSFM at 1×10^5^ cells/ml in a 24 well dish. The next day cells were scratch-wounded and incubated for 24 h in (**A**) SSFM, (**B**) 0.01 ng/ml TGFβ1, (**C**) 0.1 ng/ml TGFβ1, (**D**) 1.0 ng/ml TGFβ1, or (**E**) imaged at time zero. Bar=200 μm. **F**: Using T-Scratch software, percent cell migration into the wound margin at 24 h compared to time zero was calculated. Each condition was compared to SSFM, *p-value <0.05, **p-value <0.01. Experiments were repeated at least three times with similar results.

HCFs were scratch-wounded under SSFM conditions (-) and migration rates were compared to HCFs treated with exogenously added TGFβ1: 0.01 ng/ml, 0.1 ng/ml, and 1 ng/ml (Figure 2A-E). Percent cell migration was determined using T-Scratch software (Figure 2F) and summarized in Table 1. All TGFβ1concentrations tested decreased cell migration rates compared to the endogenous TGFβ1 in SSFM, however differences between SSFM and addition of 0.01 ng/ml were slight and were not statistically significant. The affect of TGFβ1 on cell proliferation was also tested using BrdU staining. However, after 24 h in culture, no difference in cell proliferation was found (data not shown).

**Table 1:** Summary of wound healing data after TGFβ1 treatment.

** **	**TGFβ1**			
**Data summary**	**-**	**0.01 ng/ml**	**0.1 ng/ml**	**1.0 ng/ml**
% Cell migration into the wound	86.6±2.3	68.0±9.0	31.9±5.6	12.6±3.6
% Cells with α-SMA stress fibers	2.2±1.4	36.1±4.4	90.2±8.0	85.3±11.3
Cell area (pixel intensity 1000×)	7.4±0.9	10.5±1.4	17.1±2.2	16.9±1.2
% Leading-edge cells with p38MAPK nuclear exclusion	11.9±6.7	18.7±9.9	62.7±13.3	74.8±6.4
% Leading-edge cells with SMAD 2/3 concentrated in nuclei	3.0±3.0	8.4±8.0	75.2±18.7	82.3±11.6

**Figure 3:**
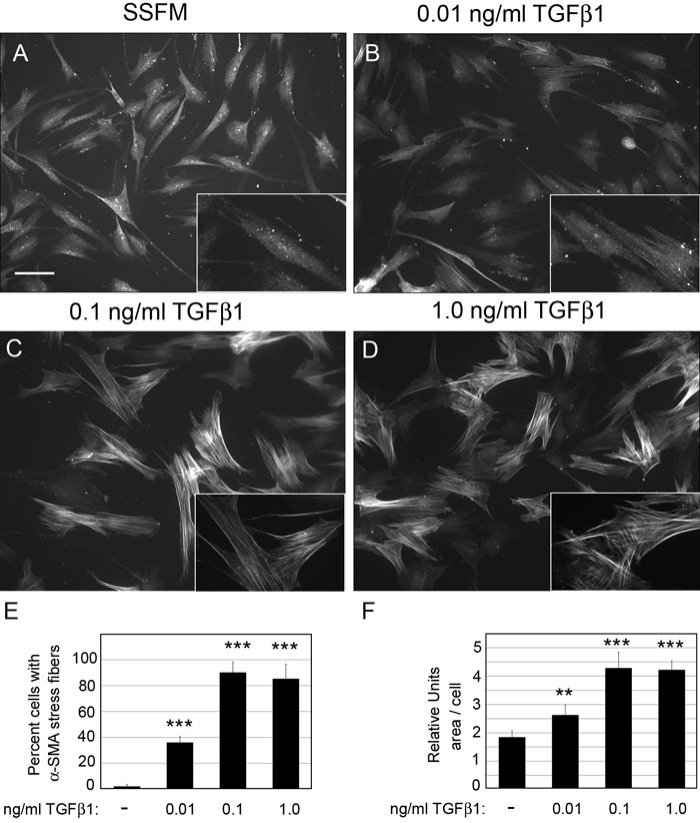
Increasing TGFβ1 concentration promotes fibrotic markers. HCFs were seeded on collagen in SSFM at 1×10^4^ cells/ml on coverslips in a 24 well dish in (**A**) SSFM, (**B**) 0.01 ng/ml TGFβ1, (**C**) 0.1 ng/ml TGFβ1, or (**D**) 1.0 ng/ml TGFβ1. After 72 h (**A**-**D**) were immunostained for α-SMA. Bar=50 μm. For each condition, cells containing organized α-SMA stress fibers were counted (**E**) and using MetaMorph Analysis the relative cell area was quantified (**F**). For analysis greater than 100 cells per experiment were analyzed. Each condition was compared to SSFM, **p-value <0.01, ***p-value <0.001. Experiments were repeated at least three times with similar results.

**Figure 4:**
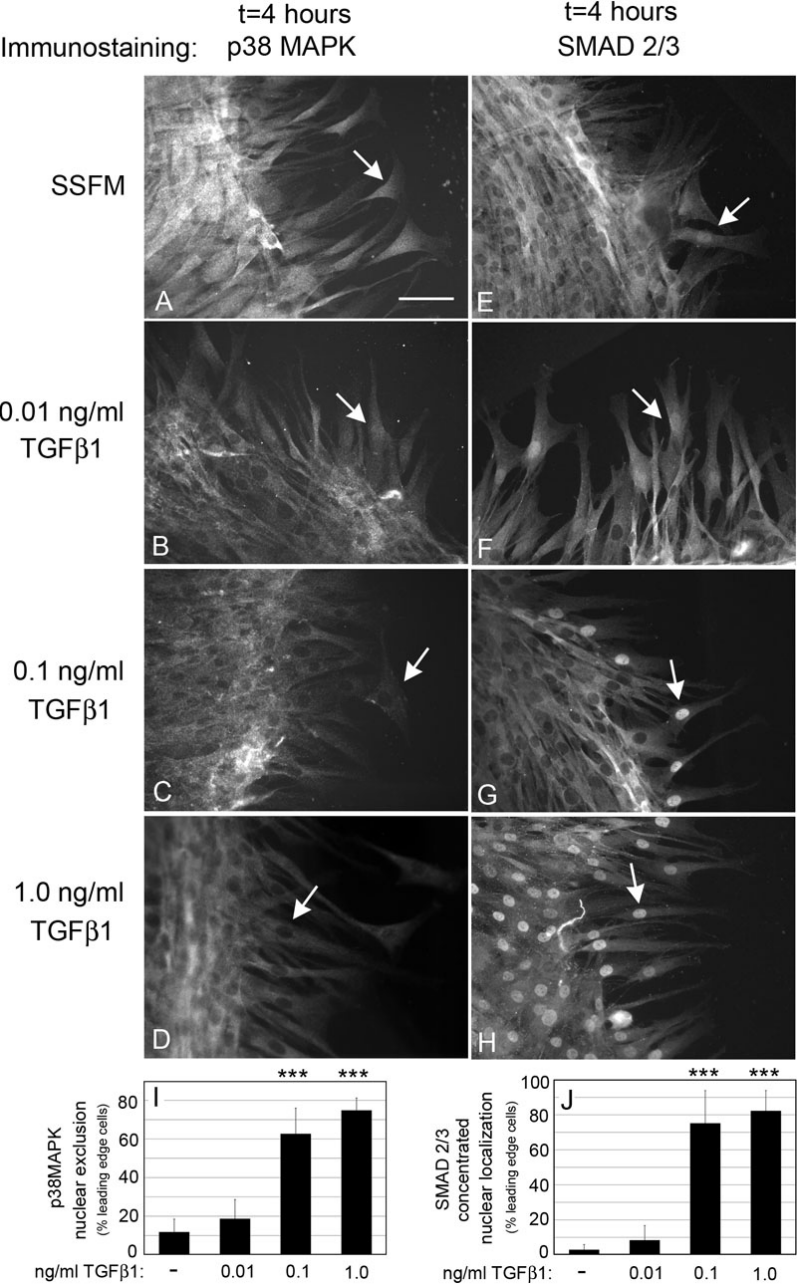
Increasing TGFβ1 concentrations results in a loss of nuclear p38MAPK. HCFs were seeded on collagen in SSFM at 1×10^5^ cells/ml in a 24 well dish. The next day cells were scratch-wounded and incubated in (**A**, **E**) SSFM, (**B**, **F**) 0.01 ng/ml TGFβ1, (**C**, **G**) 0.1 ng/ml TGFβ1, or (**D**, **H**) 1.0 ng/ml TGFβ. After 4 h cells were fixed and immunostained for p38MAPK (**A**-**D**) or SMAD 2/3 (**E**-**H**). Bar=50 μm. Arrows point to nuclei in which we detected either the nuclear localization or nuclear exclusion of p38MAPK and SMAD 2/3. The percent of cells in which p38MAPK was excluded from nuclei of the leading edge cells is shown in (**I**). The percent of cells with SMAD 2/3 concentrated in nuclei in the leading edge cells is shown in (**J**). Image J software was used for quantification. Each condition was compared to SSFM. ***p-value <0.001. Experiments were repeated at least three times with similar results.

HCFs were untreated (-), or treated with 0.01 ng/ml TGFβ1, 0.1 ng/ml TGFβ1, or 1.0 ng/ml TGFβ1. This table summarizes the data from Figure 2 (% cell migration into the wound), Figure 3 (% cells with α-SMA stress fibers and cell area), Figure 4 (% of cells with p38MAPK detected outside the nuclei in leading edge cells and % of cells with SMAD 2/3 concentrated in the nuclei in leading edge cells).

### Increasing TGFβ1 concentration generates fibrotic markers

Since TGFβ is known to promote fibrotic markers, we next investigated the impact of TGFβ concentration on myofibroblast differentiation as characterized by α-SMA stress fiber organization and increased cell area. HCFs were treated with increasing concentrations of TGFβ1 and after 72 h, cells were fixed and immunostained for α-SMA (Figure 3 A-D). As expected, the number of cells with α-SMA stress fibers increased with TGFβ1 concentration (Figure 3E and Table 1). Furthermore, using Metamorph analysis software, we found that increasing TGFβ1 concentrations resulted in a corresponding increase in their area compared to cells grown without adding TGFβ1 (Figure 3F). Of note is that 1.0 ng/ml and 0.1 ng/ml promote similar phenotypic changes in α-SMA organization and increased cell area suggesting that 0.1 ng/ml is a threshold concentration for promoting a fibrotic response. Addition of 0.01 ng/ml TGFβ1 also generated myofibroblasts after 3 days in culture but approximately 60% fewer myofibroblasts were visualized after treatment with 0.01 ng/ml compared to the two higher concentrations. As predicted, HCFs treated with TGFβ neutralizing antibody had no α-SMA stress fibers (data not shown).

### TGFβ1 concentration affects p38MAPK and SMAD 2/3 activation

Activation of p38MAPK promotes cell migration and regenerative wound healing in epithelial and endothelial corneal cells [11-13]. In contrast, activation of SMAD 2/3 is correlated with fibrotic wound healing [20]. Immunocytochemical detection of nuclear versus cytoplasmic localization of p38MAPK and SMAD 2/3 is an effective method to detect their activation only at the leading edge since their activated forms are localized to the nucleus. Thus, to determine the affect of TGFβ1 on p38MAPK and SMAD 2/3 activation, HCFs were seeded at confluence and scratch wounded in the presence of either SSFM alone, or increasing concentrations of TGFβ1. Nuclear localization of p38MAPK and SMAD 2/3 in leading edge cells was analyzed at several time points, from 1 to 8 h after wounding. At 4 h, changes in nuclear localization of p38MAPK and SMAD 2/3, in migrating cells was easily quantified. In both cases, activation is visualized by translocation to the nucleus. In SSFM (Figure 4A) and 0.01 ng/ml TGFβ1 (Figure 4B), p38MAPK was activated as indicated by its translocation to the nucleus in the leading edge cells (arrows). This is in contrast to 0.1 ng/ml TGFβ1 (Figure 4C) and 1.0 ng/ml TGFβ1 (Figure 4D) in which p38MAPK was excluded from nuclei (arrows). Data from multiple images of only leading edge cells were quantified for p38MAPK nuclear exclusion (Figure 4 I). Importantly, these data show that addition of 0.01 ng/ml TGFβ1 closely resembles that of the endogenous levels of TGFβ for the activation of p38MAPK suggesting that it is a key to promoting cell migration. In contrast the two higher concentrations inhibited p38MAPK activation. These data are supported by western blots for phospho-p38MAPK and p38MAPK after scratch wounding (Panel  **A**, in Appendix 1).

We next analyzed SMAD 2/3 activation. As predicted, SMAD 2/3 nuclear localization increased with TGFβ1 concentration (Figure 4E-H, arrows). However a low level of SMAD 2/3 activation is compatible with cell migration since the leading edge cells have detectable SMAD 2/3 in the nucleus compared to the nuclear exclusion in the cells behind the leading edge (Figure 4E-G). Quantification of leading edge cells that have discrete SMAD 2/3 localization to the nucleus is shown in Figure 4J. Data from Figure 1, Figure 2, Figure 3, and Figure 4 are summarized in Table 1. Next we sought to determine if activation of p38MAPK and SMAD 2/3 is necessary for cell migration.

### P38MAPK nuclear localization is necessary for cell migration

To assess the importance of p38MAPK activation and SMAD 2/3 to cell migration, HCFs were seeded at confluence and scratch wounded in the presence of specific inhibitors to p38MAPK and SMAD 2/3. Four h after wounding, cells were fixed and immunostained for p38MAPK and SMAD 2/3. Other cultures of scratch wounded cells were incubated with inhibitors for 18 h and imaged the next day for wound closure.

**Figure 5:**
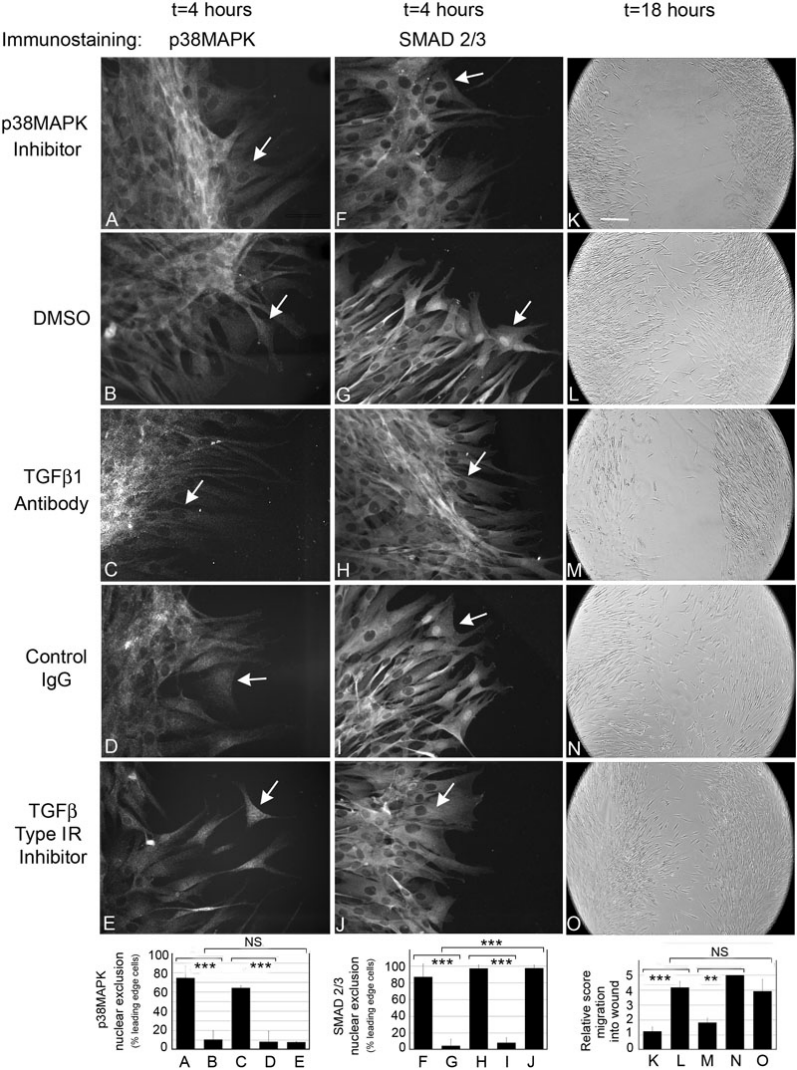
Nuclear p38MAPK localization is necessary for HCF migration. HCFs were seeded on collagen in SSFM at 1×10^5^ cells/ml in a 24 well dish. The next day cells were scratch-wounded and incubated in (**A**, **F**, **K**) p38MAPK inhibitor, 10 μM SB202190, (**B**, **G**, **L**) DMSO, (**C**, **H**, **M**) TGFβ1 antibody, (**D**, **I**, **N**) Control IgG, or (**E**, **J**, **O**) TGFβ RI (ALK5) inhibitor 10 μM SB431542. After 4 h cells were fixed and immunostained for p38MAPK (**A**-**E**) or SMAD 2/3 (**F**-**J**). Arrows denote the nuclei of leading edge cells in which p38MAPK and SMAD 2/3 were either localized or excluded (Bar=50 μm) or after 18 h cells were imaged (**K**-**O**). Bar=200 μm. DMSO is the control for addition of SB202190 or SB431542. Quantification of all data are shown in the bar graphs below the images. Left to right: Exclusion of p38MAPK from the nucleus in leading edge cells, exclusion of SMAD 2/3 from the nucleus in leading edge cells, cell migration into the wound. Nuclear localization was counted using Image J software. Two non-biased people scored cell migration, 0 (less migration) to 5 (most migration). **p-value <0.01, ***p-value <0.001. NS=not significant. Experiments were repeated at least three times with similar results.

The p38MAPK inhibitor (SB202190) prevented translocation of p38MAPK to the nucleus (Figure 5A) and also inhibited cell migration after scratch wounding (Figure 5K), demonstrating that preventing activation of p38MAPK inhibits cell migration. Since phosphorylation of SMAD 2/3 by p38MAPK is necessary for full activation, SMAD 2/3 nuclear translocation was also prevented [21,22] (Figure 5F). DMSO control cultures shown in Figure 5B,G,L, were similar to cells in SSFM alone (Figure 4A,E). To determine if blocking all TGFβ1 signaling could prevent TGFβ-mediated activation of p38MAPK, neutralizing antibody to TGFβ1 was added. We found that TGFβ1 antibody prevented activation of p38MAPK (Figure 5C) and SMAD 2/3 (Figure 5H), as well as cell migration (Figure 5M). As expected, cells that were treated with control IgG (Figure 5D,I,N) demonstrated nuclear immunostaining and wound closure rates similar to that seen in cells in SSFM alone (Figure 4A,E and Figure 1D). These data are supported by western blots for phospho-p38MAPK and p38MAPK after scratch wounding (Panel  **B**, in Appendix 1).

Next, we assessed the importance of SMAD 2/3 activation to wound closure. The SB431542 inhibitor at 10 μM prevents activin receptor-like kinase (ALK 4,5,7) and TGFβRI signaling (SMAD 2/3 activation) but does not inhibit p38MAPK activation [23]. In cells treated with 10 μM SB431542, p38MAPK was still localized to the nucleus in the leading edge cells (Figure 5E), but SMAD 2/3 was excluded from the nucleus (Figure 5J), and cells migrated at rates similar to controls (DMSO; Figure 5O). Since SMAD 2/3 is excluded from the nucleus and cells still migrate, supports the hypothesis that a low level of SMAD 2/3 activation is not necessary for cell migration. These data are quantified in bar graphs below the images in Figure 5. Left to right: Exclusion of p38MAPK from the nucleus in leading edge cells, exclusion of SMAD 2/3 from the nucleus in leading edge cells, cell migration into the wound.

Taken together, the data presented in Figure 4, Figure 5, and Appendix 1 demonstrate that TGFβ1-mediated activation of p38MAPK is necessary for cell migration and wound closure in vitro. Furthermore, that addition of a low concentration of TGFβ1 such as 0.01 TGFβ1 (additive to endogenous TGFβ) promotes p38MAPK activation.

## Discussion

### Non-healing after LASIK

Although LASIK has restored clear vision to millions of people, the post-LASIK cornea remains acellular and unhealed and thus there is a need to promote cell repopulation into the unhealed cornea [4,5]. It is possible that the lack of cell repopulation after LASIK is because LASIK remodeling of the stroma alters the ECM in a way that may inhibit cell migration from the non-wounded peripheral cornea into the wounded central cornea. It is also possible that, since the LASIK cut intersects the epithelium only at the edge of the flap, pro-migratory cytokines originating in the cut epithelium may not reach the flap bed. Our in vitro study shows that endogenous TGFβ promotes cell migration. However, the fact that post-LASIK wounds do not heal, suggests that endogenous TGFβ is not impacting wound closure post-LASIK. Thus, we investigated if adding a low concentration of TGFβ1 (equal to the levels of endogenous TGFβ) may be useful in promoting cell migration. We demonstrate that TGFβ1 at a low concentration (0.01 ng/ml) is similar to endogenous TGFβ for promoting cell migration, suggesting that in the absence of cell migration (and perhaps endogenous TGFβ), addition of this low concentration of TGFβ could substitute for endogenous TGFβ. A recent study by Mi et al. [24] aimed at increasing flap adhesion after a “LASIK-like cut” tested TGFβ1 as well as other cytokines for their ability to promote corneal wound healing. After a LASIK cut of bovine corneas, the corneas were excised placed in organ culture and treated for 4 weeks with a cytokine: TNFα, IL-1, Fas ligand, or TGFβ1. Addition of these cytokines resulted in greater flap adherence but the corneas did not remain transparent. Based on our data, the lowest concentration of TGFβ1 that Mi et al. [24] tested, 0.1 ng/ml, would generate a significant myofibroblast response. At this concentration, we report that 90% of all cells are myofibroblasts. Consistent with our findings, the authors predicted that a lower concentration could be useful in increasing flap adhesion without generating corneal haze.

### The impact of TGFβ1 concentration on p38MAPK and SMAD 2/3 signaling

TGFβ binds to the TGFβ Receptors I and II, serine/threonine kinases that initiate downstream signaling pathways. Our data support the finding that TGFβ (ligand) concentration differentially affects p38MAPK and SMAD 2/3, signaling pathways downstream of TGFβ1. Previous studies have demonstrated that pathway activation also depends on receptor expression, receptor kinase activity, and expression of receptor binding partners [25,26]. We investigated the importance of TGFβ concentration to p38MAPK activation and its effect on human corneal fibroblast wound healing in vitro because of reports demonstrating that p38MAPK activation is necessary for cell migration in other cell types [11-13,27]. Consistent with previous studies, we found p38MAPK activation in actively migrating cells. Our findings however add new insights into the TGFβ1-mediated regulation of p38MAPK since a concentration dependence of TGFβ on p38MAPK was revealed: Whereas low concentrations of TGFβ1, endogenous and (0.01 ng/ml), induced p38MAPK activation, 0.1 ng/ml and 1.0 ng/ml TGFβ prevented activation of p38MAPK. The biphasic data for low and high concentrations of TGFβ1 suggests that TGFβ receptor occupancy initiates different signals that regulate p38MAPK activation. Furthermore, these data support our hypothesis that addition of 0.01 ng/ml TGFβ1 could be used to stimulate cell migration in vivo.

SMAD 2/3 activation by TGFβ was also investigated. SMAD’s translocation to the nucleus is often used as a marker for the presence of active TGFβ and thus served as an important control for visualizing and verifying the effect of increasing concentrations of active TGFβ1. We found that as expected for a classic dose dependent result, increasing concentrations of TGFβ1 led to greater nuclear concentration of SMAD 2/3, which correlated with reduced migration by cells at the wound edge. However, a low level of SMAD 2/3 translocation to the nucleus was visualized even under the most migratory conditions (SSFM and 0.01 ng/ml TGFβ1), suggesting that SMAD 2/3 activation may be necessary for cell migration. However, the finding that cells migrated after treatment with the SMAD 2/3 inhibitor, SB431542, suggested that this is not the case. In contrast, others have shown that TGFβ-mediated inhibition of cell proliferation is dependent on SMAD 2/3 activation [21]. Thus perhaps, the minimal SMAD 2/3 translocation to the nucleus prevents cell proliferation as the cells are actively migrating.

#### Conclusion

Together our data suggest that addition of a low concentration of TGFβ may be useful for promoting human corneal fibroblast migration into a non-healing wound, without generating a large fibrotic response. These in vitro data lay the groundwork for future in vivo studies that will assess the effects of low levels of TGFβ on flap adhesion and corneal clarity.

## Appendix 1. p38MAPK activation demonstrated by western blot.

HCFs were seeded at confluence in SSFM on collagen in 100 mm plates. The next day cells were scratch-wounded (grids were used to generate consistent scratching) and reagents were added. After 4 h, cells were lysed with RIPA buffer with protease and phosphatase inhibitors. Lysates were western blotted with Phospho-p38MAPK antibody or p38MAPK antibody. Relative Pixel Intensity (RPI) was calculated for each band.  **A**: No TGFβ (-) or plus TGFβ, 0.01, 0.1, 1.0 ng/ml). SSFM is 100%. Each condition was compared to SSFM,*p value <0.05. **B**: Lane 1, p38MAPK Inhibitor (SB202190); Lane 2, DMSO; Lane 3, TGFβ1 antibody; Lane 4, Control IgG; Lane 5, TGFβRI inhibitor (SB431542). Control IgG is 100%. p value<0.05. Experiments were repeated 3 times with similar results. To access the data, click or select the words “Appendix 1.” This will initiate the download of a compressed (pdf) archive that contains the file.
